# Early Mucosal Sensing of SIV Infection by Paneth Cells Induces IL-1β Production and Initiates Gut Epithelial Disruption

**DOI:** 10.1371/journal.ppat.1004311

**Published:** 2014-08-28

**Authors:** Lauren A. Hirao, Irina Grishina, Olivier Bourry, William K. Hu, Monsicha Somrit, Sumathi Sankaran-Walters, Chris A. Gaulke, Anne N. Fenton, Jay A. Li, Robert W. Crawford, Frank Chuang, Ross Tarara, Maria L. Marco, Andreas J. Bäumler, Holland Cheng, Satya Dandekar

**Affiliations:** 1 Department of Medical Microbiology & Immunology, University of California, Davis, Davis, California, United States of America; 2 Department of Molecular and Cellular Biology, University of California, Davis, Davis, California, United States of America; 3 Department of Biochemistry and Molecular Medicine, University of California, Davis, Davis, California, United States of America; 4 Department of Primate Medicine, California National Primate Center, Davis, California, United States of America; 5 Department of Food Science and Technology, University of California, Davis, Davis, California, United States of America; Emory University, United States of America

## Abstract

HIV causes rapid CD4+ T cell depletion in the gut mucosa, resulting in immune deficiency and defects in the intestinal epithelial barrier. Breakdown in gut barrier integrity is linked to chronic inflammation and disease progression. However, the early effects of HIV on the gut epithelium, prior to the CD4+ T cell depletion, are not known. Further, the impact of early viral infection on mucosal responses to pathogenic and commensal microbes has not been investigated. We utilized the SIV model of AIDS to assess the earliest host-virus interactions and mechanisms of inflammation and dysfunction in the gut, prior to CD4+ T cell depletion. An intestinal loop model was used to interrogate the effects of SIV infection on gut mucosal immune sensing and response to pathogens and commensal bacteria *in vivo*. At 2.5 days post-SIV infection, low viral loads were detected in peripheral blood and gut mucosa without CD4+ T cell loss. However, immunohistological analysis revealed the disruption of the gut epithelium manifested by decreased expression and mislocalization of tight junction proteins. Correlating with epithelial disruption was a significant induction of IL-1β expression by Paneth cells, which were in close proximity to SIV-infected cells in the intestinal crypts. The IL-1β response preceded the induction of the antiviral interferon response. Despite the disruption of the gut epithelium, no aberrant responses to pathogenic or commensal bacteria were observed. In fact, inoculation of commensal *Lactobacillus plantarum* in intestinal loops led to rapid anti-inflammatory response and epithelial tight junction repair in SIV infected macaques. Thus, intestinal Paneth cells are the earliest responders to viral infection and induce gut inflammation through IL-1β signaling. Reversal of the IL-1β induced gut epithelial damage by *Lactobacillus plantarum* suggests synergistic host-commensal interactions during early viral infection and identify these mechanisms as potential targets for therapeutic intervention.

## Author Summary

The loss of intestinal CD4+ T cells in chronic HIV infection is associated with impaired immune responses to pathogens, aberrant immune activation, and defects in the gut epithelial barrier. While much is known about the pathogenesis of HIV in chronic disease, less is known about the defects that occur prior to gut CD4+ T cell depletion and whether these defects alter host interactions with pathogenic and commensal bacteria. Using a non-human primate model of HIV infection, we examined the immune and structural changes in the gastrointestinal tract 2.5 days following SIV infection. Paneth cells, in immediate proximity of SIV infected immune cells, generated a robust IL-1β response. This IL-1β response correlated with defects in epithelial tight junctions and preceded the IFN-α response, which is characteristic of innate antiviral immune responses. Despite this inflammatory environment, we did not observe defects in mucosal immune responses to pathogenic or commensal bacteria. In fact, commensal bacteria were able to dampen the IL-1β response and ameliorate tight junction defects. Our study highlights the importance of the gut epithelium in HIV infection, not just as a target of pathogenesis but also the initiator of immune responses to viral infection, which can be strongly influenced by commensal bacteria.

## Introduction

Chronic inflammation and disease progression in HIV infection is attributed to dysfunction in the structure of the intestinal epithelial barrier as well as impairment of the mucosal immune response resulting in increased microbial translocation [1]–[3], dysbiosis of the gut microbiome [4]–[6], and enteric opportunistic infections [7]. Incomplete recovery of gut homeostasis, despite antiretroviral therapy, contributes to the persistence of immune activation in HIV infected patients [8]–[10]. Studies in HIV infected patients and SIV infected non-human primates have shown massive dissemination of viral infection in the gut mucosa during the primary acute stage of infection leading to severe and rapid CD4+ T cell depletion [11]–[14], which persists through all stages of infection [15], [16]. In contrast, CD4+ T cell loss is progressive in peripheral blood and lymph nodes. Loss of mucosal Th17 CD4+ T cell subset coincides with epithelial barrier disruption and is linked to increased microbial translocation and chronic immune activation [17], [18]. Although immune dysfunction following mucosal CD4+ T cell loss is well described, it is not known whether HIV can alter mucosal function and epithelial integrity prior to and independent of CD4+ T cell depletion *in vivo*. Further, our understanding of mucosal resident cells that are early responders to the virus and their inflammatory signaling networks is limited.

The intestinal epithelium is functionally diverse. In addition to the digestive and absorptive functions, it plays a critical role in microbial sensing and innate antimicrobial response [19]. Secretory lineages of the intestinal epithelium produce antimicrobial products such as mucins by Goblet cells and defensins and inflammatory cytokines by Paneth cells [20]. Expansion of Paneth cells during chronic SIV infection has highlighted its important role in imparting innate defense in gut mucosa during chronic SIV infection [21]. Although the Paneth cell response to microbial pathogens is well investigated, there is no information about their response to pathogens during early HIV and SIV infections and viral pathogenesis.

There is increasing evidence that viral infections can alter the host-commensal relationship [22]. HIV and SIV induced changes in the gut microenvironment may have a profound effect on the mucosal response to incoming enteric pathogens as well as local commensal bacteria. To assess the early changes in mucosal responses induced by SIV infection, use of an *in vivo* intestinal model is essential, as *in vitro* cell culture studies fail to replicate the complex cellular interactions and anaerobic microenvironment of the gut. We developed the simian ligated intestinal loop model, which most closely recapitulates the anaerobic gut microenvironment. By directly injecting bacteria into the intestinal lumen, this model facilitates the capture of the *in vivo* dynamics between microbes, the gut epithelium, and immune cell populations during the viral infection [17].

In the present study, we investigated the earliest effects of SIV, prior to acute mucosal CD4+ T cell depletion, on epithelial barrier integrity and mucosal immune response to pathogenic (*Salmonella enterica* serovar Typhimurium, *S*. Typhimurium) and non-pathogenic (*Lactobacillus plantarum*, *L. plantarum*) bacteria *in vivo*. Our findings showed that the gut epithelium was the initial target of viral pathogenesis, as evidenced by impaired expression and disorganization of epithelial tight junction proteins, which were correlated to increased expression of interleukin-1β (IL-1β). We identified Paneth cells as the dominant source of the early innate IL-1β immune response. At this time-point, no defects in mucosal immune response to either pathogenic or commensal bacteria were observed. In fact, mucosal exposure to *L. plantarum* rapidly dampened SIV-induced inflammation through the inhibition of the NF-κB pathway. Our study identified, for the first time, Paneth cells as an initial source of gut inflammation and IL-1β signaling during early viral infection. In addition, anti-inflammatory and epithelial repair effects of *L. plantarum* suggest the potential role of commensal bacteria in reversing the early effects of viral pathogenesis.

## Results

### Defects in the intestinal epithelium precede mucosal CD4+ T cell depletion in early SIV infection

**Figure 1:**
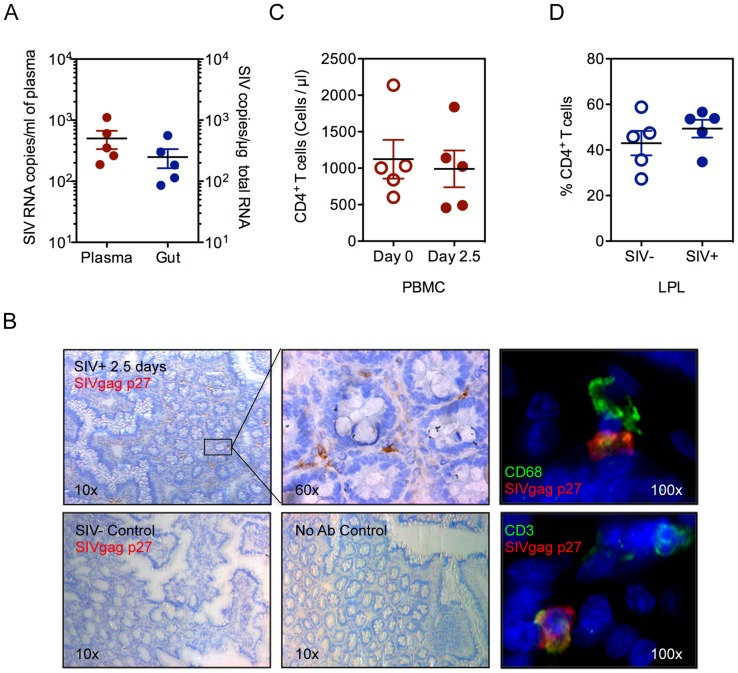
Viral infection is established in the peripheral blood and gut at 2.5 days post-SIV infection. (**A**) Viral RNA loads were detected in the blood plasma and intestinal tissue by real time PCR assay. (**B**) Presence of SIV infected cells in ileal tissue was determined by immunohistochemistry for SIVgag p27 (brown). By immunofluorescence, CD68^+^ macrophages (Top: green) and CD3^+^ T cells (Bottom: green) were positive for SIVgag p27 (red). Representative images are shown. (**C, D**) Quantification of CD4+ T cells was done by absolute count in the peripheral blood (**C**) or as a percentage of CD3+ LPLs isolated from ileal tissue (**D**) by flow cytometry.

To identify the earliest targets of the pathogenic effects of SIV infection in the gut mucosa, prior to CD4+ T cell depletion, we examined rhesus macaques at 2.5 days following SIV infection (SIV+). Viral RNA was readily detected in plasma and intestinal tissue, indicating that productive viral infection was established in both mucosal and peripheral blood compartments (**Figure 1A**). Plasma viral loads ranged from 188–1106 RNA copies/ml (502.4±166.3 copies/ml) while viral loads in intestinal tissue ranged from 86–562 SIV copies/µg total RNA (249.7±86.44 SIV copies/µg total RNA). The localization and phenotype of SIV infected cells in intestinal tissues was determined by immunohistochemistry (IHC) (**Figure 1B** **, Figure S1**). A small number of SIV-positive cells were detected, mostly in clusters near the lower crypt regions of the intestinal mucosa, and were either CD3+ T cells or CD68+ macrophages. (**Figure 1B** **, Figure S1**). There was no detectable loss of CD4+ T cells, either in the peripheral blood (baseline uninfected: 1063±262.5 and SIV+: 952.5±322.4 cells/µl) (**Figure 1C**) or in the gut mucosa (percentage range 40.99–52.72%) (**Figure 1D**). Further, no significant changes were observed in CD4+ T cell activation, in either peripheral blood or gut mucosa, as determined by HLA-DR expression (**Figure S2**).

**Figure 2:**
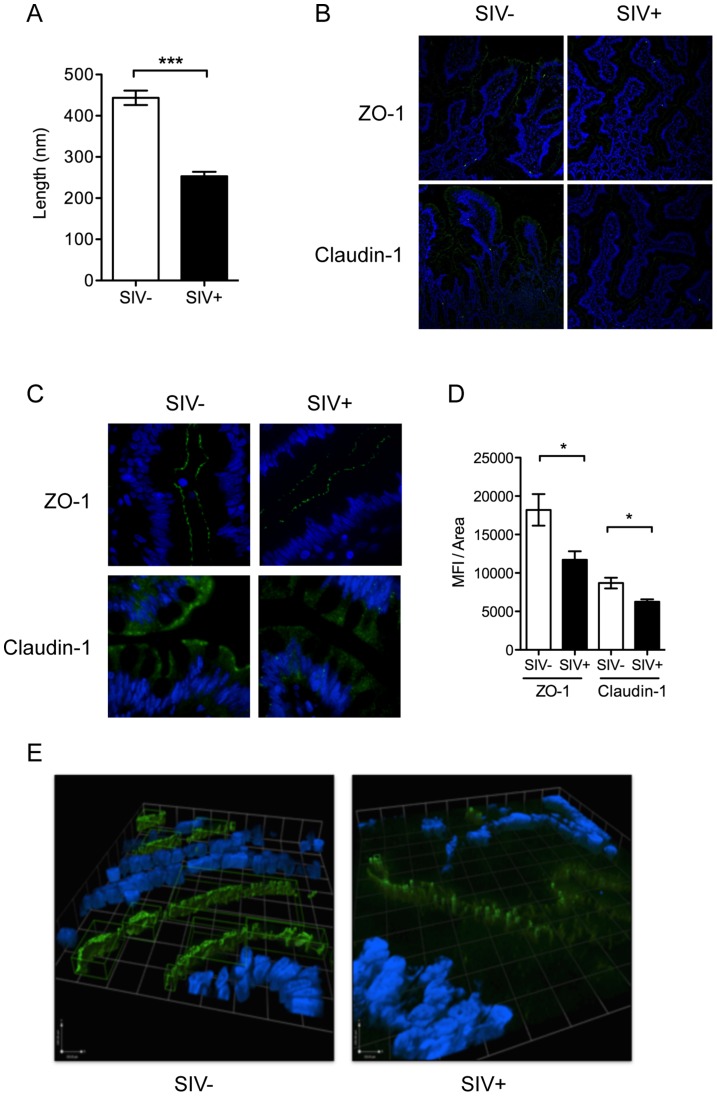
Intestinal epithelial barrier is an early target of SIV infection. (**A**) Interepithelial tight junction length was measured in ileal tissues from SIV− and SIV+ animals by EM. (**B, C**) Distribution of tight junction complex proteins, Claudin-1 and ZO-1 (green), was determined by IF at 20× (**B**) and 60× (**C**) maginification. (**D**) ZO-1 and Claudin-1 staining was quantified as the mean fluorescence intensity/area measured using Image J software. (**E**) 3D modeling of ZO-1 protein distribution showed epithelial disorganization. Regions in green indicate high fluorescence intensity of >1000 counts. * p<0.05, **p<0.01, ***p<0.001.

The rapid depletion of mucosal CD4+ T cells has been implicated in dysfunction of epithelial barriers and immune response during chronic HIV and SIV infections [16]. Despite the lack of detectable gut CD4+ T cell depletion at 2.5 days of SIV infection, we observed the onset of early defects in the gut epithelium by electron microscopy (EM). Epithelial tight junction structures were significantly shorter in SIV+ animals (253±10.73 nm) compared to uninfected controls (443.5±17.38 nm) (*P*<0.001, Mann-Whitney) (**Figure 2A**). Analysis of tight junction proteins by confocal microscopy confirmed that SIV infection also caused a significant reduction in the expression of tight junction proteins, ZO-1 and Claudin-1 (*P* = 0.027 and 0.015, respectively, Mann-Whitney) (**Figure 2B–D**). In addition, the distribution of ZO-1 was discontinuous in SIV+ animals; which may suggest an impairment of epithelial structure and organization since ZO-1 is an intracellular scaffolding protein integral to the organized assembly of epithelial tight junction complexes (**Figure 2E**). However, the reduction and restructuring of tight junction proteins during early SIV infection did not result in increased systemic microbial translocation, as determined by the levels of bacterial lipopolysaccharides (LPS) in the plasma (**Figure S3**).

### Induction of IL-1β production by Paneth cells precedes the type 1 IFN response in the gut mucosa

**Figure 3:**
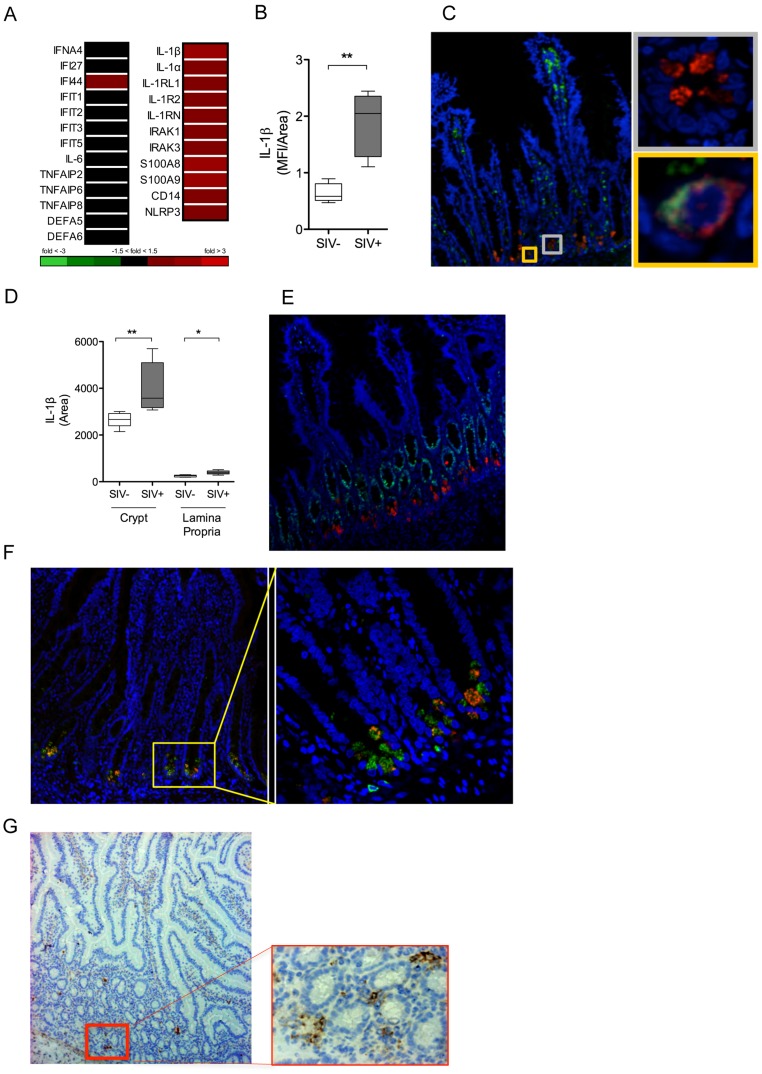
IL-1β production by Paneth cells precedes the IFN antiviral response in early SIV infection. (**A**) The induction of IFN and IL-1β pathways in the gut mucosa was identified by DNA microarray analysis of whole ileal tissue. (**B**) Mean fluorescence intensity (MFI) of IL-1β signal was quantified in the ileal mucosa. (**C**) Co-localization of IL-1β (red), CD68+ (green) and DAPI (blue) were determined by IF. Yellow inset: IL-1β^+^ CD68^+^ cell (macrophage). Grey inset: IL-1β^+^ CD68^−^ cell. (**D**) The total area of IL-1β detected in the crypt and lamina propria regions in the intestinal tissues of healthy controls and SIV infected animals was determined using Image J software. (**E, F**) Phenotypic analysis of IL-1β (red) producing cells was determined by co-staining with Ki67 (green) (**E**), a marker of proliferating cells, and lysozyme A (green) (**F**), a protein found in the granules of Paneth cells. (**G**) Localization of SIV infected cells was determined by IHC staining for SIVgag p27. All representative images are shown at 20× magnification with insets shown at 60× magnification. *P<0.05, **P<0.01, ***P<0.001.

To identify the earliest immune responses to viral infection in the gut mucosa, we performed transcriptome analysis of the intestinal tissues at 2.5 days following SIV infection using rhesus macaque specific DNA microarrays. There was no detectable increase in the expression of several known innate immune pathways or antiviral interferon (IFN) stimulated genes (ISG) in the intestinal tissues of SIV infected macaques compared to healthy controls (**Figure 3A**). In contrast, a striking induction of IL-1β expression and increased expression of IL-1β regulated genes was observed (**Figure 3A**). Evaluation of intestinal tissues by immunostaining confirmed a significant increase of IL-1β protein mean fluorescence intensity (MFI) in early SIV infection (*P* = 0.007) (**Figure 3B**).

To localize, and identify the major IL-1β expressing cells in response to SIV infection in the gut mucosa, immunohistochemical analysis was performed. The phenotype of IL-1β expressing cells was determined by the co-localization of IL-1β protein with several specific cellular markers. IL-1β was detected in the crypt epithelium as well as in lamina propria immune cells (**Figure 3C**). However, IL-1β expression in the crypt epithelium was approximately ten-fold higher than that observed in the lamina propria (**Figure 3D**). Macrophages are known to produce IL-1β following activation of the inflammasome [23]. We found that some of the IL-1β–expressing cells were positive for CD68, CD163 and CD206 expression, which served as the macrophage specific cell surface markers (**Figure 3C** **, Figure S4**). Paneth cells are differentiated, secretory cells that release defensins and antimicrobial enzymes into the intestinal lumen. Paneth cells in the intestinal tissues were identified based on their location at the base of the crypt epithelium, detectable secretory granules, absence of Ki67 cell proliferation marker (**Figure 3E**) and the presence of anti-microbial lysozyme protein in the granules (**Figure 3F**) by confocal microscopy. When we examined the localization of SIV infected cells by immunostaining for the SIV p27 antigen, we observed that the infected cells were localized in close proximity to Paneth cells in the intestinal crypts (**Figure 3G**). These findings suggested epithelial-immune cell interactions in the initial mucosal response to the virus.

### Increased IL-1β production is associated with early disruption of epithelial tight junctions

**Figure 4:**
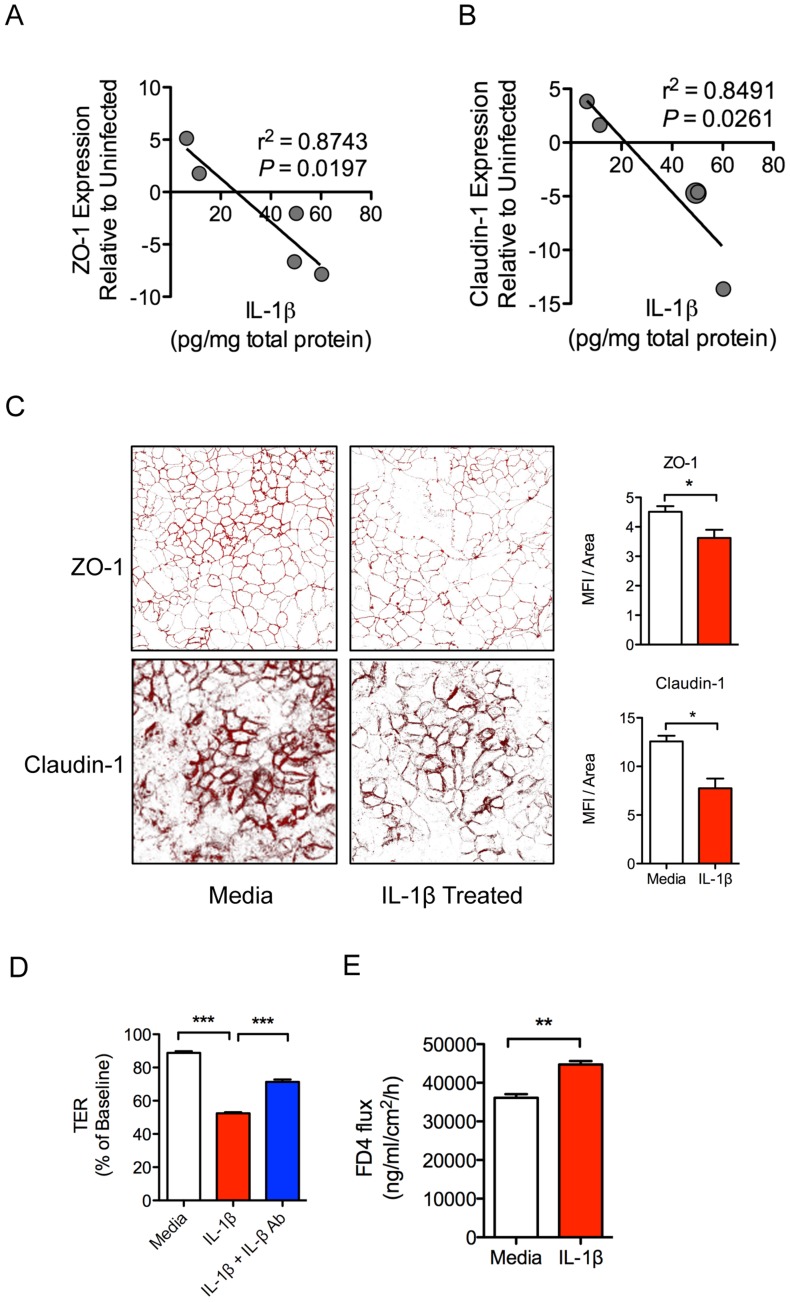
IL-1β causes intestinal epithelial barrier dysfunction *in vivo* and *in vitro*. (**A, B**) Pearson correlations between IL-1β protein levels in intestinal tissues from SIV infected animals with gene expression levels of tight junctions proteins ZO-1 (**A**) and Claudin-1 (**B**). (**C**) Caco2 epithelial cell monolayers were used in transwell experiments to determine the effects the basolateral addition of IL-1β on ZO-1 and Claudin-1 protein expression. Representative images are shown at 63× magnification. (**D, E**) Changes in epithelial barrier integrity following the addition of IL-1β to Caco2 cells were measured by transepithelial electrical resistance (TER) (**D**) and increased flux of 4 kDa-FITC dextran (FD4) across the cells (**E**). *P<0.05, **P<0.01, ***P<0.001.

IL-1β production in intestinal tissue during early SIV infection was negatively correlated with the expression of tight junction proteins ZO-1 (r^2^ = 0.874; *P* = 0.019) and Claudin-1 (r^2^ = 0.849; *P* = 0.026) (**Figure 4A, B**). These *in vivo* findings were validated by *in vitro* epithelial cell culture studies, where basolateral IL-1β treatment of Caco2 intestinal epithelial cells induced significant decreases in ZO-1 and Claudin-1 protein expression (**Figure 4C**), and increased permeability as measured by a decrease in trans-epithelial electrical resistance (TER) (**Figure 4D**). Addition of an IL-1β blocking antibody caused a significant rebound in TER (**Figure 4D**). To determine whether decreased TER reflected reduced barrier function, we measured 4 kDa-FITC dextran (FD4) flux, and found a significant increase in flux across the Caco2 monolayer following IL-1β treatment (**Figure 4E**). Together, these data provide compelling evidence that early IL-1β production following SIV infection plays a role in epithelial disruption.

### Gut mucosal immune responses to pathogenic bacteria at 2.5 days post-SIV infection are intact and functional

We previously reported that the depletion of CD4+ Th17 cells in chronic SIV infection impairs gut mucosal immune response to pathogenic bacteria and leads to systemic microbial translocation [17]. Therefore, we sought to determine whether the onset of functional defects in the gut mucosal immune sensing and response to pathogenic (*S*. Typhimurium) or commensal (*L. plantarum*) bacteria occurred immediately upon viral exposure and prior to CD4+ T cell depletion. We utilized the ligated ileal loop model that allows for the real-time interrogation of mucosal immune responses to luminally-injected bacteria in an *in vivo* setting (**Figure S5**).

**Figure 5:**
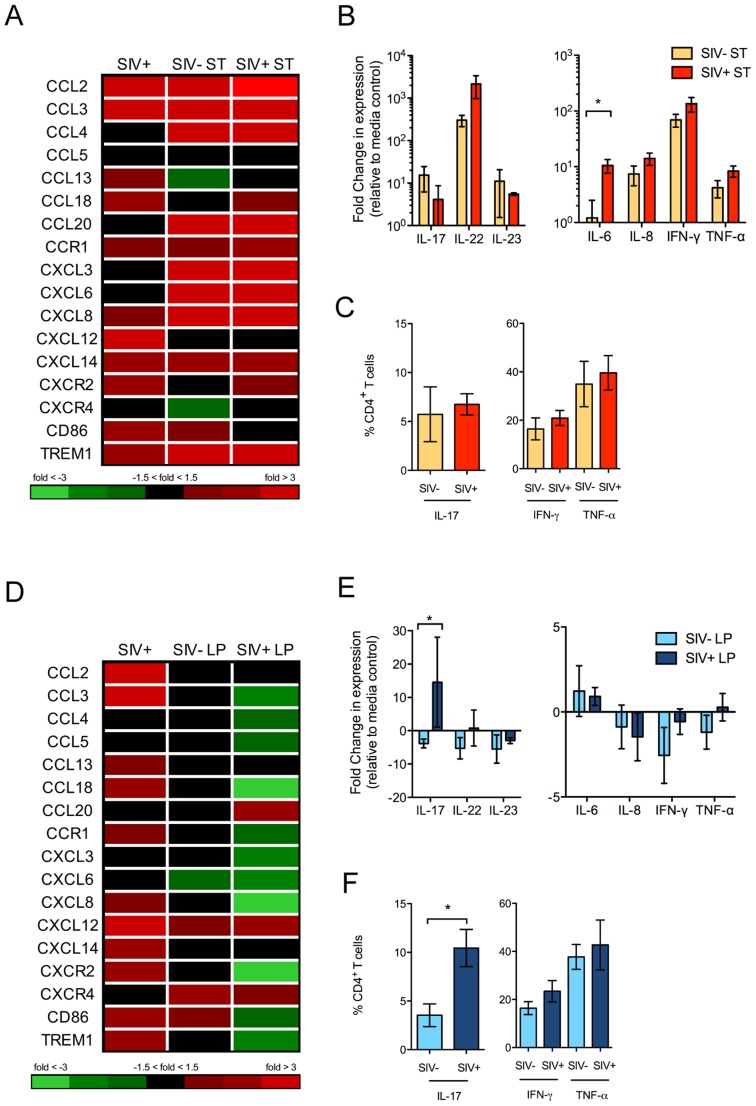
Distinct gut mucosal immune responses to pathogenic and commensal bacteria during early SIV infection. (**A, D**) Changes in the gene expression patterns in response to *S.* Typhimurium (ST) (**A**) and *L. plantarum* (LP) (**D**) inoculation were detected by DNA microarray analysis. Fold changes in the transcription of chemokines compared to uninfected controls are shown. (**B, E**) Production of Th1 and Th17 cytokines in ST (**B**) and LP (**E**) inoculated intestinal loop tissues was determined by RT-PCR. (**C, F**) Th1 and Th17 populations present in the ileal lamina propria of ST (**C**) or LP (**F**) inoculated loops was determined by intracellular cytokine staining following *ex vivo* mitogen stimulation. The data are shown as means ± SEM. *P<0.05, **P<0.01, ***P<0.001.

There was no systemic translocation of either *S*. Typhimurium or *L. plantarum* to peripheral sites from the lamina propria following intraluminal injection of bacteria into ileal loops of SIV infected animals (**Figure S6A, B**). Both live *S*. Typhimurium and *L. plantarum* could be detected in the lumen following incubation, however only pathogenic *S*. Typhimurium could be detected in the lamina propria (**Figure S6 A–D**). Further, to determine whether the presence of SIV infection in the gut mucosa was sufficient to induce aberrant mucosal immune response to *S.* Typhimurium, gene expression analysis was performed using DNA microarrays. A robust increase in mucosal gene expression associated with chemotaxis of neutrophils and monocytes, Th17 responses, and proinflammatory cytokines was detected in SIV-negative healthy controls in response to *S.* Typhimurium (**Figure 5A**). The transcriptional profiles in SIV+ macaques in response to *S.* Typhimurium were comparable to those present in SIV-negative controls with an exception for IL-6, whose expression was significantly elevated (*P* = 0.02) (**Figure 5B**). The percentages of Th17 and Th1 CD4+ T cell subsets in *S*. Typhimurium inoculated loops were not altered or depleted at 2.5 days post-SIV infection (**Figure 5C**). Thus, SIV infection did not dampen the ability of the gut immune system to mount a marked response against *S*. Typhimurium.

### *L. plantarum* reduces SIV-induced gut inflammation

In contrast to the effects of *S*. Typhimurium, inoculation with *L. plantarum* in the intestinal loops of SIV-negative control animals had a minimal effect on the mucosal gene expression profiles (**Figure 5D**). However, intestinal loops from SIV+ animals had a significant change in the gene expression profiles in response to *L. plantarum* compared to control loops without *L. plantarum*. This included striking downregulation of genes involved in inflammation and cell trafficking of monocytes and neutrophils (CD86, TREM1 and CXCL8) and upregulation of genes associated with epithelial repair and tissue remodeling.

An exception to the general downregulation of chemokines in the intestinal loops following *L. plantarum* inoculation was the increased expression of CXCR4, CXCL12 and CCL20. The CXCR4-CXCL12 axis has been utilized by several pathogens, including HIV, for entry and invasion [24]. CCL20 is a chemokine involved in the recruitment of Th17 cells [25]. We found that there was a significant increase in IL-17 transcript levels (*P* = 0.03, Mann-Whitney) (**Figure 5E**) as well as a marked increase in the frequency of Th17 cells in intestinal loops of SIV+ animals compared to SIV-negative controls (*P* = 0.02, Mann-Whitney) (**Figure 5F**). In comparison, no changes were observed in inflammatory cytokine expression or Th1 cells between these two groups.

**Figure 6:**
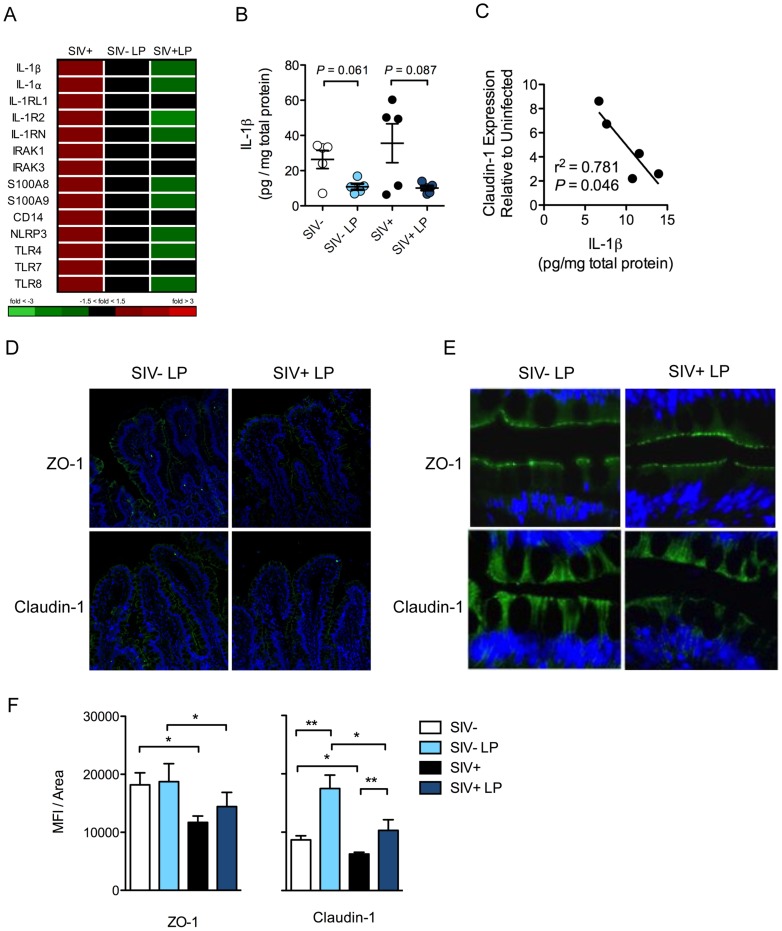
*L. plantarum* suppresses mediators of inflammation and enhances epithelial tight junction integrity. (**A**) Changes in IL-1β related gene expression due to early SIV infection, and the subsequent effect on mucosal responses to *L. plantarum* (LP), was assessed by DNA microarray analysis. (**B**) IL-1β protein expression in LP inoculated and media control tissue was measured by ELISA. (**C**) Correlation between Claudin-1 mRNA expression and IL-1β protein expression in the LP inoculated tissues of SIV+ animals. (**D**, **E**) Expression of tight junction proteins: ZO-1 (Green) and Claudin-1 (Green) were measured by immunofluorescence. Representative images are shown at 20× (**D**) and 60× (**E**). (**F**) The expression of ZO-1 and Claudin-1 was quantified and are shown as mean fluorescence intensities (MFI) ± SEM. *P<0.05, **P<0.01, ***P<0.001.

*L. plantarum* inoculation also resulted in the decreased expression of IL-1β and other genes involved in IL-1β production and signaling (**Figure 6A**). There was a similar reduction in IL-1β protein levels in both SIV+ and SIV-negative animals in response to *L. plantarum* (*P* = 0.087 and 0.061, respectively) (**Figure 6B**). The decrease in IL-1β protein expression in response to *L. plantarum* inoculation was inversely correlated to Claudin-1 mRNA expression, which was increased in SIV+ animals following *L. plantarum* inoculation (r^2^ = 0.781, *P* = 0.046) (**Figure 6C**). *L. plantarum* also significantly increased Claudin-1 protein expression *in vivo* in both SIV+ and SIV-negative animals (**Figure 6D–F**) compared to the controls (without *L. plantarum*) (**Figure 1B–D**) (*P* = 0.008 and 0.007, respectively). Our data suggest that *L. plantarum* has the potential to reverse IL-1β-associated epithelial barrier injury caused during early stages of SIV infection.

### *L. plantarum* reduces IL-1β induced epithelial damage through NF-κB inhibition

**Figure 7:**
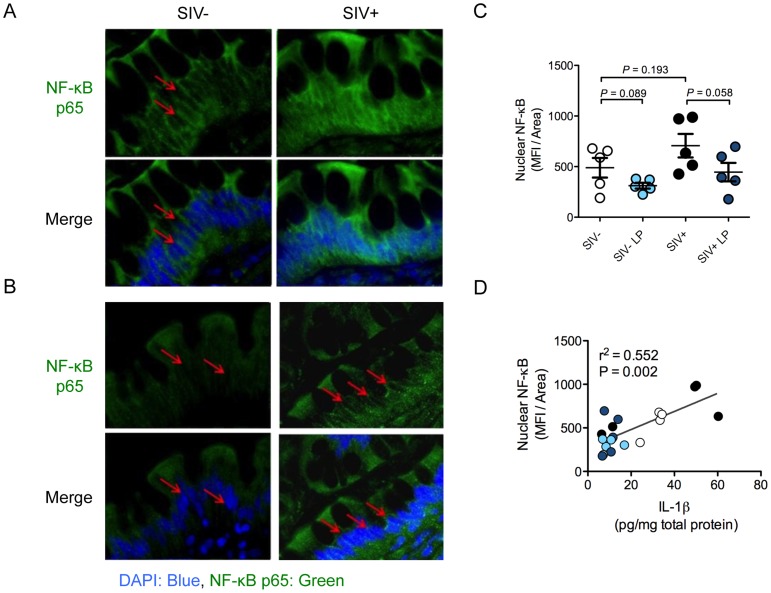
*L. plantarum* reduces IL-1β induced epithelial damage by inhibition of NF-κB activation. (**A, B**) Activation of the NF-κB pathway was assessed by the nuclear localization (DAPI: blue) of p65 (green) as determined by immunofluorescence. Representative images from intestinal loops from SIV+ animals and SIV-negative controls are shown at 60× magnification (**A**). Red arrows indicate areas of p65 nuclear localization. (**B**) Representative images from LP inoculated loops at 60× magnification. Red arrows indicate nuclei devoid of p65 staining. (**C**) Nuclear NF-κB in LP inoculated intestinal loops compared to control loops was quantified using Image J software and is expressed as mean fluorescence intensity/area. (**D**) IL-1β protein expression in SIV+ and SIV-negative intestinal loops correlated with the level of nuclear translocation of NF-κB, both in media control loops (SIV−: white and SIV+: black circles) and LP inoculated loops (SIV−: light blue and SIV+: dark blue circles).

We sought to elucidate the mechanism by which IL-1β expression in the gut mucosa was reduced by *L. plantarum*. NF-κB is a transcription factor that regulates IL-1β expression and whose activation is characterized by its nuclear translocation [26]. NF-κB activation was detected by its nuclear localization using immunostaining. SIV infection alone caused an increase in the level of nuclear NF-κB translocation and but this increase was not significant (*P* = 0.193) (**Figure 7A**). Following *L. plantarum* inoculation, a trend of reduction in nuclear NF-κB protein localization was observed in both SIV+ animals and SIV-negative controls (*P* = 0.058 and 0.089, respectively) (**Figure 7B, C**). The levels of nuclear NF-κB positively correlated with the levels of IL-1β observed in all animals (r^2^ = 0.596, *P* = 0.008), regardless of the SIV infection status or presence of *L. plantarum* (**Figure 7D**). These observations suggest that *L. plantarum* is able to reduce IL-1β protein expression through the inhibition of the NF-κB nuclear translocation in the intestinal epithelium.

## Discussion

Our study, for the first time, reports that Paneth cells are early sensors of virally infected immune cells in the intestinal mucosa. Their inflammatory response is mediated through robust IL-1β signaling, with profound implications on early tissue damage. Thus, Paneth cells play a critical role in the induction of gut inflammation during the early stages of viral infection, prior to the depletion of CD4+ T cells. To our knowledge, this is the first description of IL-1β production by Paneth cells.

While the mechanism by which Paneth cells sense and respond to pathogenic bacteria is well characterized, our understanding of their response to HIV infection is limited [20]. We found that SIV infected cells were localized in close proximity of the crypt epithelium, potentially exposing Paneth cells to viral antigens or inflammatory cytokines released by the infected cells. In HIV infection, virus has been shown to induce NLRP3-inflammasome expression and IL-1β production in myeloid cells [27]. Though we cannot definitively attribute the induction of IL-1β to a specific stimulus, NLRP3 expression was increased in the gut mucosa suggesting potential involvement of an NLRP3-inflammasome mediated pathway in Paneth cells during SIV infection. Our findings highlight the need for future investigations to determine the mechanisms of Paneth cell sensing and response to viral infections and their role in the induction of host innate response to HIV.

We previously reported an increased expression of enteric defensins in Paneth cells during primary and chronic SIV infection that correlated with viral loads [21]. The loss of defensin accumulation in these cells correlated with disease progression and opportunistic infections. In the present study, we did not observe an increase in enteric defensin gene expression at 2.5 days of SIV infection. This suggests that the IL-1β response precedes the upregulation of defensin expression in Paneth cells. Similarly, we did not detect a significant increase in the expression of IFN-α or IFN stimulated genes (ISG). The type 1 IFN response is critical in the early containment of viral replication [28]. However, this involves the recruitment of plasmacytoid dendritic cells to the gut mucosa and may require higher levels of viral replication than occurs at 2.5 days following SIV infection [28]–[30]. Thus, IL-1β production by Paneth cells represents a local response to SIV infection at a time point when viral presence is low in the intestinal mucosa, and may critically impact innate immune cell subsets such as macrophages and innate lymphoid cells (ILC), which express IL-1β receptors [31], [32].

Inflammatory cytokines have been shown to disrupt epithelial barrier integrity [33]. Exposure to IL-1β increased permeability in intestinal epithelial cell cultures by decreasing epithelial tight junction protein expression [34]–[36]. Increased IL-1β expression at 2.5 days of SIV infection negatively correlations with expression of tight junction components in our study, suggesting that IL-1β initiates intestinal epithelial barrier defects. Other inflammatory cytokines, such as IFN-γ and TNF-α, have also been shown to cause disruption of epithelial cell tight junctions *in vitro* [37]. However, we did not detect an upregulation of IFN-γ or TNF-α expression by transcriptome analysis *in vivo*, suggesting that these cytokines might not contribute significantly towards intestinal epithelial changes during early infection. HIV envelope protein gp120 was shown to induce defects in epithelial tight junctions, only when added apically to epithelial cell cultures. No effects were observed when gp120 was added basolaterally [38]. This mechanism is unlikely to play a role in epithelial integrity defects observed in our study, given that the few SIV infected immune cells detected were localized to the basolateral side of the intestinal epithelium.

In chronic SIV disease, epithelial barrier disruption has been shown to lead to increased microbial translocation. However, the changes in the intestinal epithelial barrier that occur during early viral infection did not result in systemic dissemination of bacteria and microbial products. This discrepancy is likely due to the preservation of mucosal CD4+ T cells in early infection, as our previous study had shown that the depletion of Th17 cells, in chronic SIV infection, results in the increased dissemination of pathogenic *S*. Typhimurium [17].

The ability of the mucosal immune system to rapidly eradicate pathogens while maintaining tolerance to commensal bacteria is critical to the maintenance of intestinal homeostasis. The occurrence of aberrant host immune responses to commensal bacteria has been reported during chronic inflammatory conditions such as inflammatory bowel diseases (IBD) [39] and recently in acute *Toxoplasma gondii* infection [22]. Aberrant inflammatory response to commensal bacteria by peripheral monocytes of individuals with chronic HIV infection has been reported [40]. It is not known whether acute HIV infection might obfuscate the host's ability to distinguish between pathogen and commensals. Aberrant immune responses to commensal bacteria during chronic HIV infection may be attributed to increased microbial translocation [2], immune activation of antigen presenting cells [40], [41], and increased TLR2 and TLR4 expression [42], [43]. However, there have been no known studies that have interrogated gut mucosal immune responses to commensal bacteria in the context of early HIV infection. In our study, SIV infected animals had enhanced inflammatory responses to *S.* Typhimurium, compared to SIV-negative controls, but showed no significant changes in the response to *L. plantarum*. Thus, in early SIV infection, the host maintains its ability to distinguish pathogenic and commensal bacteria and mount the proper immune response.

We found that *L. plantarum* rapidly induced intestinal epithelial repair in SIV infected macaques through anti-inflammatory effects that were evident by decreased expression of IL-1β and inflammatory chemokines. Previous studies reported on the ability of *Lactobacillus* species to enhance epithelial barrier integrity via tight junction regulation [44]–[46]. Lactobacilli are known to regulate the NF-κB signaling cascade in both intestinal epithelial and antigen presenting cells [47], [48]. In the current study, significant correlations were found linking disruption of epithelial tight junctions, induction of IL-1β levels, NF-κB activation and the ability of *L. plantarum* to downregulate these pathologic processes. This raises a possibility of exploiting of *L. plantarum* to intervene the early mucosal-viral interactions that may influence gut inflammation. In addition to its anti-inflammatory effects, we observed enhanced recruitment of Th17 cells in response to *L. plantarum*, mostly likely due to the induction of CCL20 expression. This recruitment of Th17 cells may have a role in epithelial repair. Our findings suggest a supportive role of *L. plantarum* in overcoming SIV-induced gut inflammation and epithelial tight junction disruption. However, unintended consequences of an *L. plantarum* probiotic therapeutic adjuvant may include increased viral replication through recruitment of virus-susceptible Th17 cell targets and viral dissemination through the induction of the CXCR4-CXCL12 axis. Our findings raise an important consideration in the development of probiotic therapies for HIV infection and highlight the need for a better characterization of probiotic bacterial functions and effects [49], [50].

In summary, our study has identified the gut epithelium, specifically Paneth cells, as a site of sensing and response of viral infection and an inducer of gut inflammation through IL-1β signaling during early SIV infection. The ability of *L. plantarum* to modulate NF-κB activation and ameliorate epithelial defects makes it an attractive therapeutic adjuvant. These results highlight the importance of the trialogue between the epithelium, immune cells, and commensal organisms in the restoration and protection of the intestinal mucosa [51]. By further understanding the mechanisms that underlie the host/microbiota relationship in health and HIV disease, we can capitalize on their evolved synergy while identifying gaps in mucosal defenses that can be fortified through therapy.

## Materials and Methods

### Animal experiments

Ten male rhesus macaques ranging from 3 to 6 years of age (tested negative for SIV, STLV, Salmonella) underwent ligated ileal loop surgery (Table S1). Five macaques were inoculated intravenously with 1000 TCID_50_ of SIVmac251 for 2.5 days, while five healthy, uninfected macaques served as negative controls. Animals were anesthetized and underwent ileal loop surgery as previously described [17]. Briefly, a laparotomy procedure was performed to expose the ileum before the ligation of 13 loops with an average of 5 cm in length, leaving 1-cm spacer loops in between. One ml of either stationary phase culture containing 1×10^9^ colony-forming units (CFU) of wild type S. Typhimurium (IR715) or *L. plantarum* (WCFS1) was injected directly into the lumen of the ileal loops. Loops inoculated with sterile LB or MRS broth served as media controls. Each animal had three replicates of each inoculation and one loop that was not inoculated, and served as an injection control (**Figure S5**). All intestinal loops were collected at 5 hours (hr) following the bacterial administration. Six mm punch biopsies were collected from each intestinal loop as well as the jejunum, mesenteric lymph node, liver, and spleen for bacteriology as previously described [17]. Bacteriological data were obtained to confirm injected bacteria survival following 5 hours of incubation inside the intestinal lumen. All animals were housed at the California National Primate Research Center.

### Ethics statement

This study was carried out in strict accordance with the recommendations of the Public Health Services (PHS) Policy on Humane Care and Use of Laboratory Animals. All animals were housed at the California National Primate Research Center. All animal procedures were performed according to a protocol approved by the Institutional Animal Care and Use Committee of the University of California, Davis (protocol number: 17287). Appropriate sedatives, anesthetics and analgesics were used during handling and surgical manipulations to ensure minimal pain, suffering, and distress to animals. Furthermore, housing, feeding and environmental enrichment were in accord with recommendations of the Weatherall report. Animals were euthanized in accordance with the American Veterinary Medical Association (AVMA) Guidelines for the Euthanasia of Animals (Section 2.3)

### Viral load measurements

SIV RNA loads in plasma and gut tissue samples were determined by real-time reverse transcription-PCR (RT-PCR) assay as previously described [52]. Briefly, viral RNA was isolated from 1 µg of tissue and reverse transcribed to cDNA using Supercript III. SIV gag sequences were detected using a previously published Taqman system using an Applied Biosystems ViiA 7 detection system, and data were analyzed with ViiA 7 RUO software (Applied Biosystem). The data was extrapolated against a standard curve and viral RNA copies/µg of total RNA or RNA copies/ml plasma were calculated and presented.

### Bacteriology

Six-mm biopsy punches were collected from the mesenteric lymph nodes and spleen. Biopsy punches were homogenized, serially diluted, and plated on LB + Carbenicillin (100 µg/ml) agar and MRS + Rifampicin (50 µg/ml) agar plates. To detect *S*. Typhimurium and *L. plantarum* in the lumen of ileal loops, 100 µl of luminal fluid was homogenized, serially diluted, and plated. Similarly, 1 mL of whole blood was homogenized and 100 µl was serially diluted and plated to determine the systemic dissemination of injected bacteria.

### Plasma LPS assay

Plasma samples were diluted 1∶5 in endotoxin-free water and incubated for 15 minutes at 70°C to inactivate plasma proteins [2]. LPS was then measured using the Limulus Amebocyte Assay (Lonza) according to the manufacturer's protocol. Samples were run in triplicate and LPS levels were quantified using a standard curve after background subtraction.

### Transmission Electron Microscopic (TEM) analysis of intestinal epithelial tight junctions

Intestinal loop tissues were embedded in Araldite/Epon resin (Electron Microscopy Sciences) and 100 nm thin sections were produced using a Leica ultramicrotome. Sections were mounted on copper grids and then post-stained with 2% uranyl acetate and 1% lead citrate. Samples were imaged under a JEOL 1230 transmission electron microscope operated at 120 kV and the micrographs were digitally recorded on a TVIPS F214 CCD camera at magnification of 8000–10000×. The step size on the CCD is 14 um and the pixel size at specimen space was calculated for each micrograph according to its magnification and the post column modification in the microscope. The lengths of tight junction were measured with program GIMP, as number of pixels spanning the adhesive plasma membrane from the micrograph and then converted into nanometer by multiplying the corresponding pixel size.

### Immunofluorescence and immunocytochemistry

Immunohistochemical analysis was performed using either frozen OCT embedded or 4% paraformaldehyde (PFA) fixed, paraffin embedded tissues. For Immunofluorescence: 5 µm sections were rehydrated and antigen retrieval (DAKO) was performed at 95°C for 30 min. Tissues were then blocked with 1% Fc blockers (Miltenyi Biotec) and 10% serum (Jackson ImmunoResearch Laboratories Inc.) for 30 min, incubated with primary antibody overnight at 4°C, followed by the secondary antibody for 1 hr at room temperature. For immunocytochemistry: 5 µm sections were fixed with cold acetone and blocked with DAKO dual endogenous enzyme block. Primary antibodies were incubated overnight at 4°C followed by development with 3,3′-diaminobenzidine (DAB). The primary antibodies were as follows: mouse monoclonal IgG1 anti-SIVmac251 Gag (clone: KK64) (NIH AIDS Reagents), rabbit polyclonal anti-human CD3 (DAKO), rabbit polyclonal anti-human ZO-1 and claudin-1 (Invitrogen), goat IgG anti-human IL-1β (R&D Systems), mouse monoclonal IgG1a anti-human CD68 (DAKO), mouse monoclonal IgG2a anti-human CD68 (Thermo Scientific), rabbit polyclonal anti-human lysozyme (DAKO), mouse monoclonal IgG1a anti-human Ki-67 (DAKO), polyclonal rabbit anti-human NF-κB p65 (Abcam), mouse anti-CD163 (clone: 10D6) (Leica Biosystems Newcastle), rabbit polyclonal anti-CD206 (Sigma-Aldrich), mouse monoclonal IgG1 anti-LTA (Santa Cruz Biotechnologies), and DifcoTM Salmonella O Antisera (BD Pharmingen). Alexa Flour 488 donkey anti-rabbit, Alex Fluor 488 goat anti-mouse IgG2a, Alexa Fluor 488 donkey anti-mouse IgG, Alexa Fluor 555 goat anti-mouse IgG1, Alexa Flour 555 donkey anti-rabbit and Alexa Fluor 555 donkey anti-goat secondary antibodies were used (Invitrogen). Isotype control was performed for IL-1β using a goat IgG UNLB (Southern Biotech). Nuclei were visualized using DAPI nucleic acid stain (Invitrogen). Images were collected using DeltaVision PersonalDV Deconvolution microscopy (Applied Precision), Leica DM IL LED microscope (Leica Microsystems) and LSM 700 microscope (Zeiss).

### Immunofluorescence image analysis

For the detection of epithelial tight junction proteins and NF-κB, gut tissues were imaged using Z-stack with 0.2 µm per section (25 sections total). These were performed in triplicate (3 slides with minimum of 30 µm distance separating tissue triplicates). An oil immersion, 60× objective (na = 1.42) was used with 2×2 binning during image acquisition. The sum of fluorescence intensity was calculated for the stack and mean fluorescence intensity (MFI) was determined. MFI of tight junction proteins within the epithelial regions of the tissue were quantified. For quantification of nuclear NF-κB, DAPI signal regions were selected and NF-κB signal within this region was analyzed. IL-1β localization was imaged using a 10×, 20× and 40× objectives. We utilized the 20× images to quantify the area of IL-1β within the crypts and lamina propria in the intestinal mucosa. Crypt epithelium was defined as epithelial cells most proximal to the basement membrane, as compared to protein in the lamina propria, which included regions of immune cells but not epithelial cells. Image J software (National Institute of Mental Health) was utilized for image processing and quantification.

### Assessment of *in vitro* effects of IL-1β on epithelial tight junctions

For *in vitro* cell culture experiments, Caco-2 cells were treated with IL-1β for 24 hr and washed twice with PBS (Invitrogen) and fixed for 15 min with an acetone/methanol solution (1/1 v/v), permeabilized with a 1% Triton X-100 solution (Sigma-Aldrich) and blocked with 3% milk in PBS for 1 hr. Cells were then incubated with primary antibodies (Claudin-1, and ZO-1) overnight at 4°C followed by an incubation with a secondary antibody (1∶200) for 1 hr. Filters were mounted on slides with coverslip using Slow fade mounting media (Invitrogen). Slide were then analysed using a LSM 700 microscope (Zeiss) and fluorescence level was quantified using Image J software.

### RNA extraction and microarray analysis

Total RNA was isolated utilizing the Qiagen RNeasy RNA isolation kit (Qiagen). Messenger RNA amplification, labeling, hybridization to rhesus macaque genome GeneChips, (Affymetrix) staining, and scanning were performed as described previously [52].

Assignment of genes to functional categories was performed through annotation of gene lists using the Affymetrix NetAFFX web interface, the DAVID (http://david.abcc.ncifcrf.gov/) annotation tool, and through literature-based classification by hand. Statistically over-represented (Fisher exact probability score <0.05) biological processes within sub-clusters were identified using Ingenuity Pathway Analysis (Ingenuity Systems Inc., Redwood City, CA).

### Measurement of host gene expression by real-time PCR

Cryopreserved tissue samples were used for real-time PCR analysis. Primer-probe pairs tested, and validated to have an amplification efficiency of >95%, comparable to that of glyceraldehyde-3-phosphate dehydrogenase (GAPDH). Primers were either obtained from Applied Biosystems (Foster City, CA) or were designed, optimized, and validated for use by the Lucy Whittier Molecular Core Lab (University of California, Davis) (**Table 1**). Relative mRNA expression levels were calculated from normalized Δ*C_T_* (cycle threshold) values and are reported as the change. In this analysis, the *C_T_* value for the housekeeping gene (GAPDH) was subtracted from the *C_T_* value of the target gene for each sample for normalization. The target gene and GAPDH amplified with the same efficiency (data not shown). The Δ*C_T_* value for the tissue sample from the calibrator was then subtracted from the Δ*C_T_* value of the corresponding tissue sample from the experimental loop (ΔΔ*C_T_*). The increase in mRNA levels in loop tissue samples of the experimental loops compared to tissue samples of baseline (calibrator) animals was then calculated as follows: increase = 2^ΔΔ*CT; decrease*^ = −(2^(Abs (ΔΔ*CT*))^) (ViiA™ 7, Applied Biosystems).

**Table 1:** RT-PCR gene primer sequences and probe IDs.

Gene	Probe ID	Forward Primer Sequence	Reverse Primer Sequence	Probe Sequence
**IL-17a**	Hs00220924_m1	–	–	–
**IL-22**	Hs00220924_m1	–	–	–
**IL-23**	Hs00900828_g1	–	–	–
**IL-6**	Rh02621719_m1	–	–	–
**IL-8**	Hs00174103_m1	–	–	–
**TNFα**	Hs00174128_m1	–	–	–
**TJP1**	Hs00543824_m1	–	–	–
**Occludin**	Hs00170162_m1	–	–	–
**Claudin 1**	Hs00221623_m1	–	–	–
**prGAPDH**	–	GCACCACCAACTGCTTAGCACC	TCTTCTGGGTGGCAGTGATG	TCGTGGGAAGGACTCATGACCACAGTCC
**prIFNγ**	–	AAGCTGACCAATTATTCGGTAACTG	AGTTCAGCCATCACTTGGATGA	TCAAATGTCCAACGCAAAGCAGTACATGA

### Cell Isolation, stimulation and flow cytometry

Lamina propria lymphocytes (LPLs) were isolated from macaque tissue as described previously [53]. Following isolation, LPLs were incubated with or without 25 ng/ml PMA and 1 µg/ml ionomycin (Sigma-Aldrich) in the presence of Golgi Plug (BD Bioscience, San Jose, CA) for 6 hours. Cells were stained with Aqua LIVE/DEAD viability dye (Invitrogen) and subsequently stained for T cell phenotype markers CD3 (SP34-2, BD Bioscience), CD4 (OKT4, eBioscience), and CD8 (RPA-T8, Biolegend). Cells were then permeabilized with CytoFix/CytoPerm (BD Bioscience) and stained for IL-17 (eBio64CAP17, eBioscience), IFN-γ (4SB3, eBioscience), and TNF-α (MAb11, eBioscience). To assess T cell activation cells were *ex vivo* stained with HLA-DR (L243, Biolegend) in addition to the previously described markers: CD3, CD4, CD8.

Cells were analyzed on a LSRII flow cytometer (BD Bioscience). A minimum of one million events was collected per sample. Data analysis was performed using FlowJo version 8.8.6 (TreeStar).

### IL-1β protein extraction and assessment

Cellular proteins were extracted from 30–50 mg of ileal tissue using RIPA buffer (Sigma) with protease inhibitor (Roche) and homogenizing the tissue by bead agitation in a MagNA Lyser (Roche). Samples were then centrifuged and supernatant was utilized for further analysis. IL-1β protein level was measured by ELISA assay (IL-1β Quantikine, R&D Systems). Data was normalized to total tissue protein, assessed by the Bradford protein assay (Biorad).

### Epithelial permeability studies

Caco2 cells (ATCC®HTB-37) were grown in MEM media (Invitrogen) supplemented with 20% fetal bovine serum (Gemini Bioproducts), 1% Antibiotic-Antimycotic (Invitrogen). Caco2 cells only from passages 20 to 30 were used. Caco2 cells were cultured (5×10^5^ cells/well) on permeable 0.4 µm polycarbonate filter membranes (Corning) until they reached confluence and a transepithelial electrical resistance (TER) higher than 1000. TER was measured using a Millicell-ERS voltohmeter (Millipore). Caco2 cells were then treated with IL-1β (Sigma-Aldrich) at the basolateral side of the membrane. TER measurements were performed just before and 24 hr after addition of IL-1β. FD4 (Sigma-Aldrich) flux across the Caco2 monolayer was assessed 24 h after IL-1β treatment. After withdrawing the media and washing the insert with HBSS, FD4 solution (500 µl; concentration 1 mg/ml) was added to the apical chamber and the fluorescent intensity of FD4 in the apical chamber was measured at 1 h by a fluorescent microplate reader (Chameleon V, Hidex).

### Statistical analysis

For comparisons of tight junction length in SIV infection a 2-tailed, unpaired t-test with Welch's correction was performed. For data from IF, real-time PCR, and flow cytometry a two-tailed Mann Whitney test was performed. Pearson correlation was utilized to determine all coefficients of determination. Data pertaining to the changes observed due to bacterial inoculation, as compared to its media control within the same study animal, a paired two-tailed T-test was performed. P-values<0.05 were considered significant (GraphPad Software).

## Supporting Information

Figure S1 **SIV-infected cells are localized near the crypt epithelium of the gut mucosa.** Representative images of immunohistochemical staining of ileal tissue from all infected macaques at 2.5 days post-infection for the SIVgag p27 protein (brown) at 10× magnification. The ileal tissue section from a healthy, uninfected animal (SIV− Control) is shown as a control.
(TIF)

Figure S2 **No increase in CD4+ T cell activation at 2.5 days post-SIV infection.** The activation status of CD4+ T cells was assessed by HLA-DR staining on cells isolated from the gut (LPL) or peripheral blood (PBMC) by flow cytometry. Activated CD4+ T cells were defined as live, CD3^+^CD4^+^CD8^−^HLA-DR^hi^ cells. The data are shown as a percentage of live CD3^+^CD4^+^CD8^−^ cells.
(TIF)

Figure S3 **No increase in plasma LPS levels at 2.5 days post-SIV infection.** Gut barrier integrity at 2.5 days of SIV infection was measured by plasma lipopolysaccharide (LPS) level in both infected and uninfected macaques. The data are shown as EU/ml of plasma ± SEM.
(TIF)

Figure S4 **Intestinal mucosal macrophages express IL-1β.** Immunofluorescence analysis was used to identify intestinal macrophages that produce IL-1β. Colocalization of IL-1β (red) and cells positive for the macrophage markers (**A**) CD206 (green) and (**B**) CD163 (green) was observed in the ileal tissues of SIV infected macaques. Representative images are shown at 20× magnification with insets shown at 60× magnification.
(TIF)

Figure S5 **Ligated intestinal loop model.** A schematic of the ligated ileal loops and un-injected 1 cm spacer loops (circles) are shown. Loops were inoculated with either *L. plantarum* (orange) or *S*. Typhimurium (blue). Loops were injected with MRS (red) or LB (green) broth as a negative control.
(TIF)

Figure S6 **Absence of systemic translocation of** ***S*** **. Typhimurium or** ***L. plantarum*** **.** Bacteriological assessments of live (**A**) *S*. Typhimurium and (**B**) *L. plantarum* were performed on the intestinal luminal content and sites peripheral to the intestinal mucosa: mesenteric lymph node (MsnLN), blood, and spleen. Immunohistochemistry was used to visualize the localization of (**C**) *S*. Typhimurium and (**D**) *L. plantarum* in the intestinal lumen and in the lamina propria of ileal loops. Representative images are shown at 20× magnification with insets shown at 60× magnification.
(TIF)

Table S1 **Animal data and clinical parameters.**
(TIF)
