# Supplementary figures and images for: Early Mucosal Sensing of SIV Infection by Paneth Cells Induces IL-1β Production and Initiates Gut Epithelial Disruption
Source: PLoS Pathog. 2014 Aug 28;10(8):e1004311. doi: 10.1371/journal.ppat.1004311 (PMC4148401; doi:10.1371/journal.ppat.1004311)

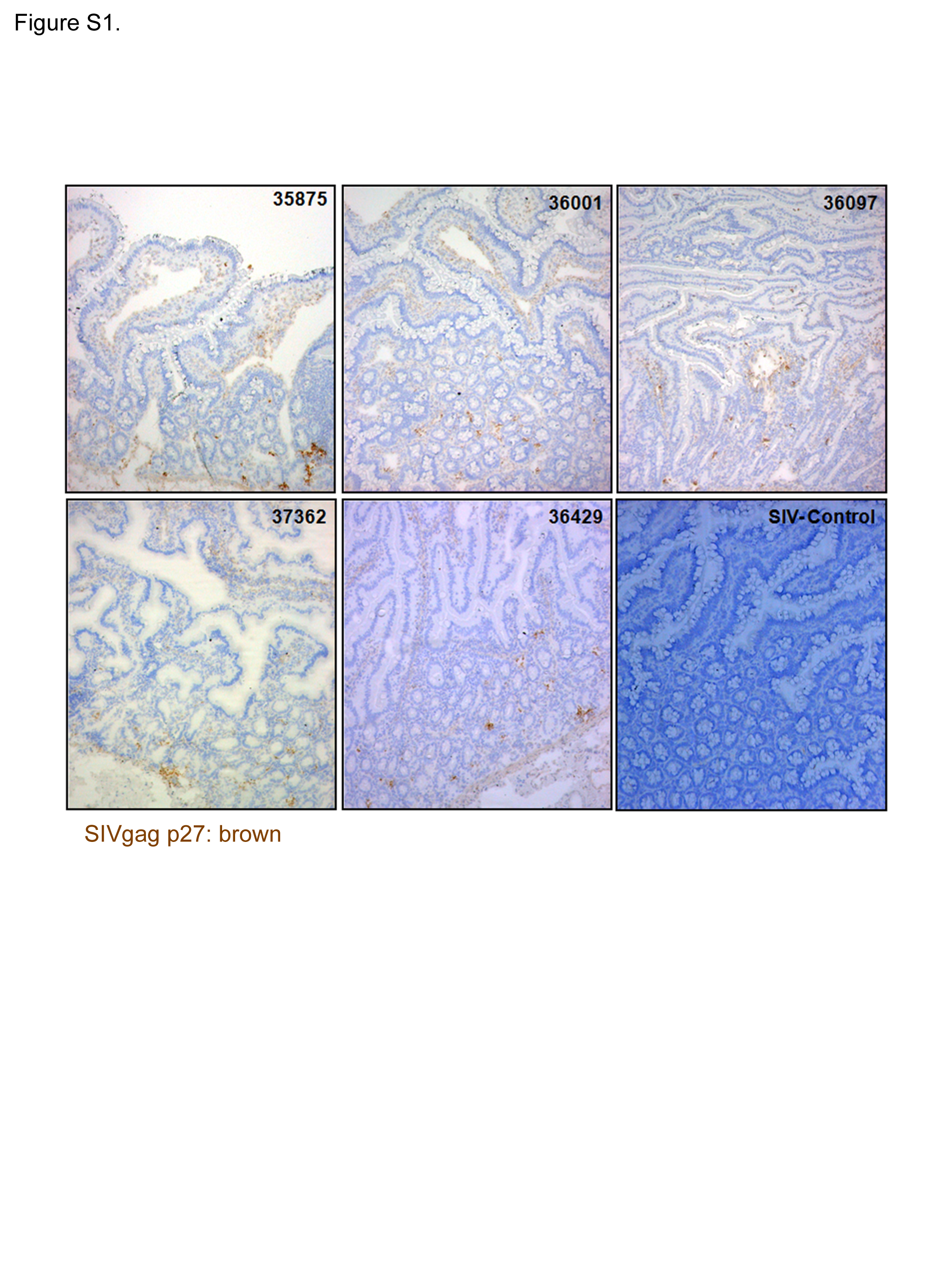

Supplement: Figure S1 — SIV-infected cells are localized near the crypt epithelium of the gut mucosa. Representative images of immunohistochemical staining of ileal tissue from all infected macaques at 2.5 days post-infection for the SIVgag p27 protein (brown) at 10× magnification. The ileal tissue section from a healthy, uninfected animal (SIV− Control) is shown as a control. (TIF) [file ppat.1004311.s001.tif]

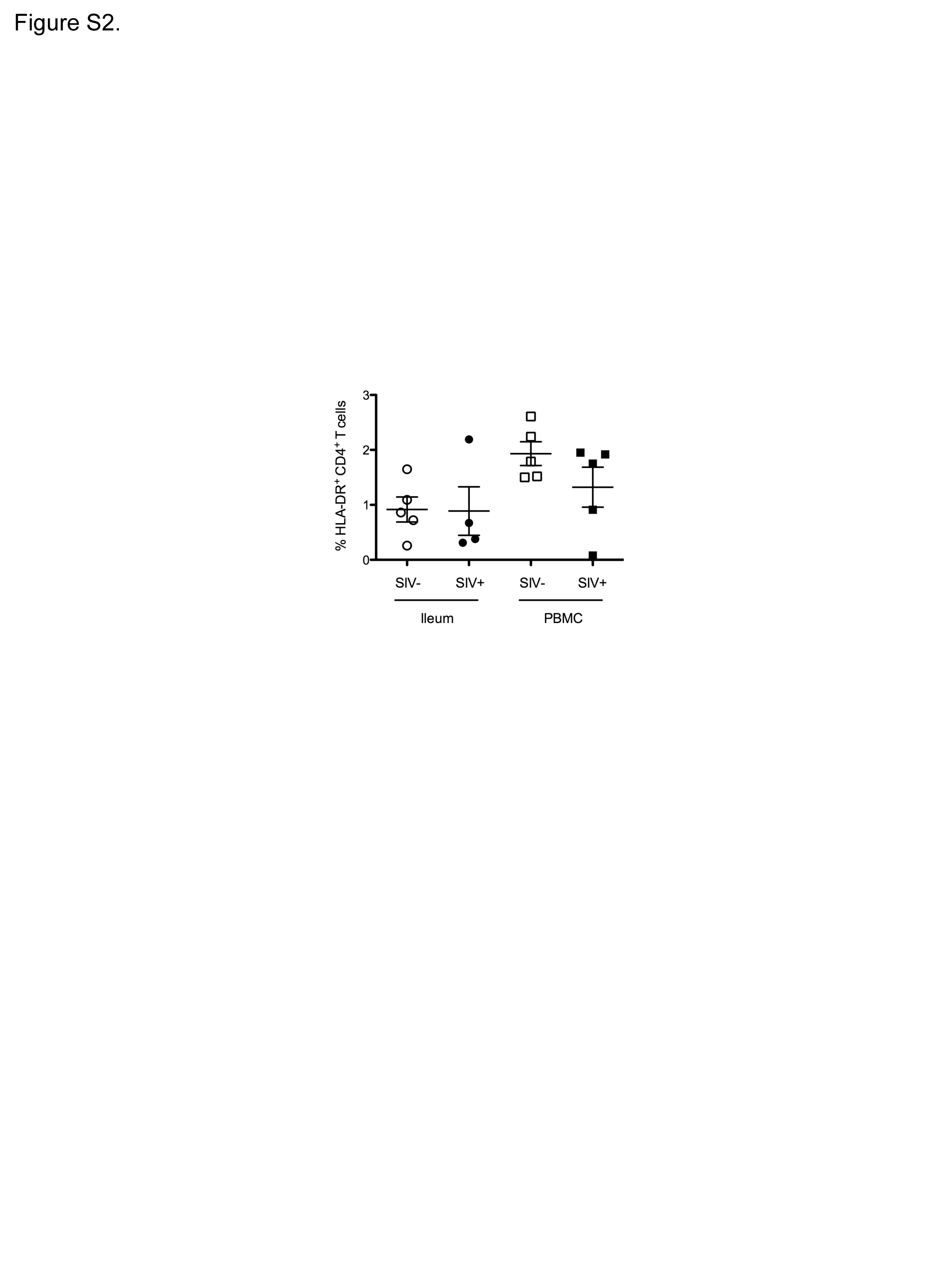

Supplement: Figure S2 — No increase in CD4+ T cell activation at 2.5 days post-SIV infection. The activation status of CD4+ T cells was assessed by HLA-DR staining on cells isolated from the gut (LPL) or peripheral blood (PBMC) by flow cytometry. Activated CD4+ T cells were defined as live, CD3+CD4+CD8−HLA-DRhi cells. The data are shown as a percentage of live CD3+CD4+CD8− cells. (TIF) [file ppat.1004311.s002.tif]

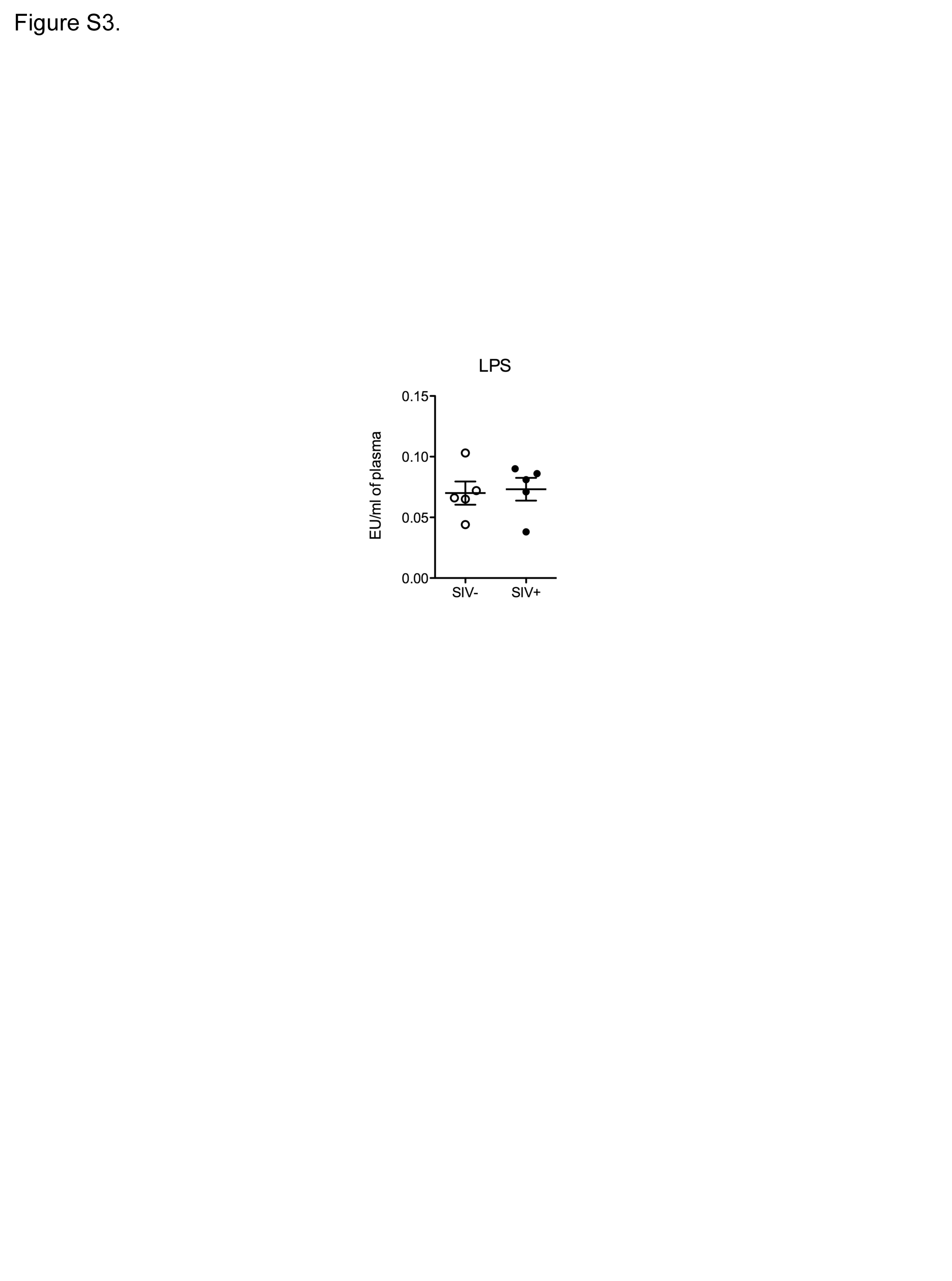

Supplement: Figure S3 — No increase in plasma LPS levels at 2.5 days post-SIV infection. Gut barrier integrity at 2.5 days of SIV infection was measured by plasma lipopolysaccharide (LPS) level in both infected and uninfected macaques. The data are shown as EU/ml of plasma ± SEM. (TIF) [file ppat.1004311.s003.tif]

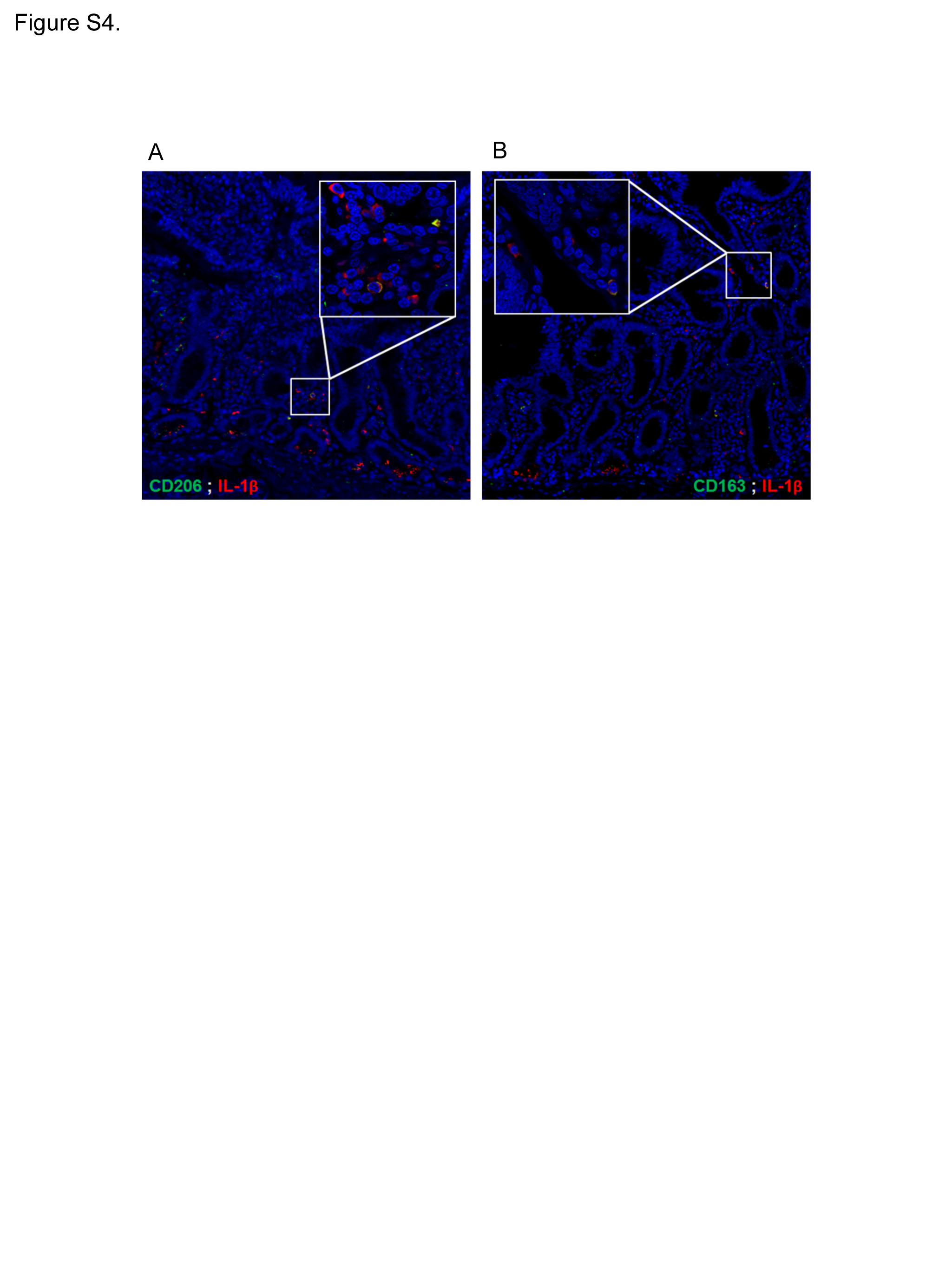

Supplement: Figure S4 — Intestinal mucosal macrophages express IL-1β. Immunofluorescence analysis was used to identify intestinal macrophages that produce IL-1β. Colocalization of IL-1β (red) and cells positive for the macrophage markers (A) CD206 (green) and (B) CD163 (green) was observed in the ileal tissues of SIV infected macaques. Representative images are shown at 20× magnification with insets shown at 60× magnification. (TIF) [file ppat.1004311.s004.tif]

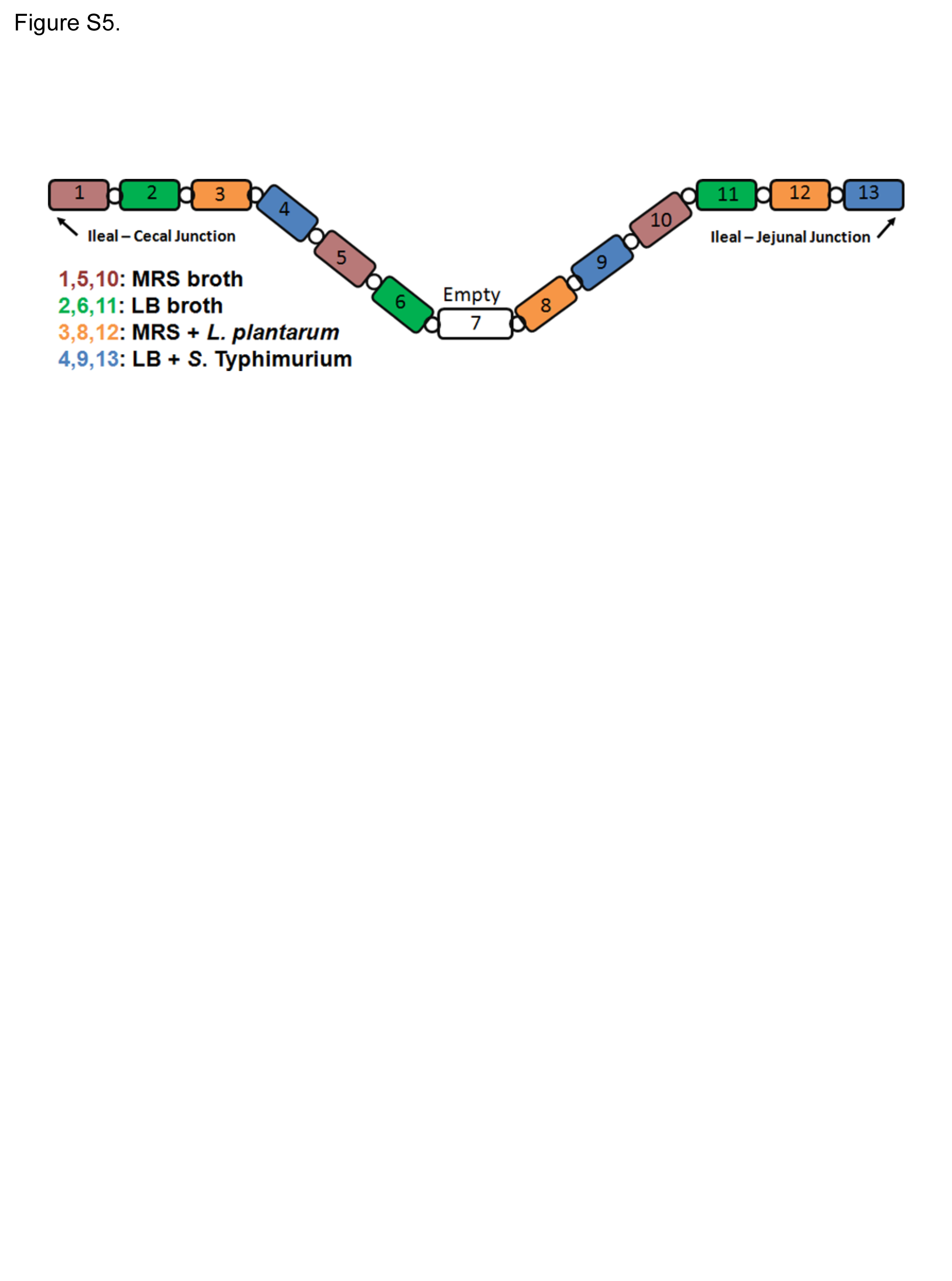

Supplement: Figure S5 — Ligated intestinal loop model. A schematic of the ligated ileal loops and un-injected 1 cm spacer loops (circles) are shown. Loops were inoculated with either L. plantarum (orange) or S. Typhimurium (blue). Loops were injected with MRS (red) or LB (green) broth as a negative control. (TIF) [file ppat.1004311.s005.tif]

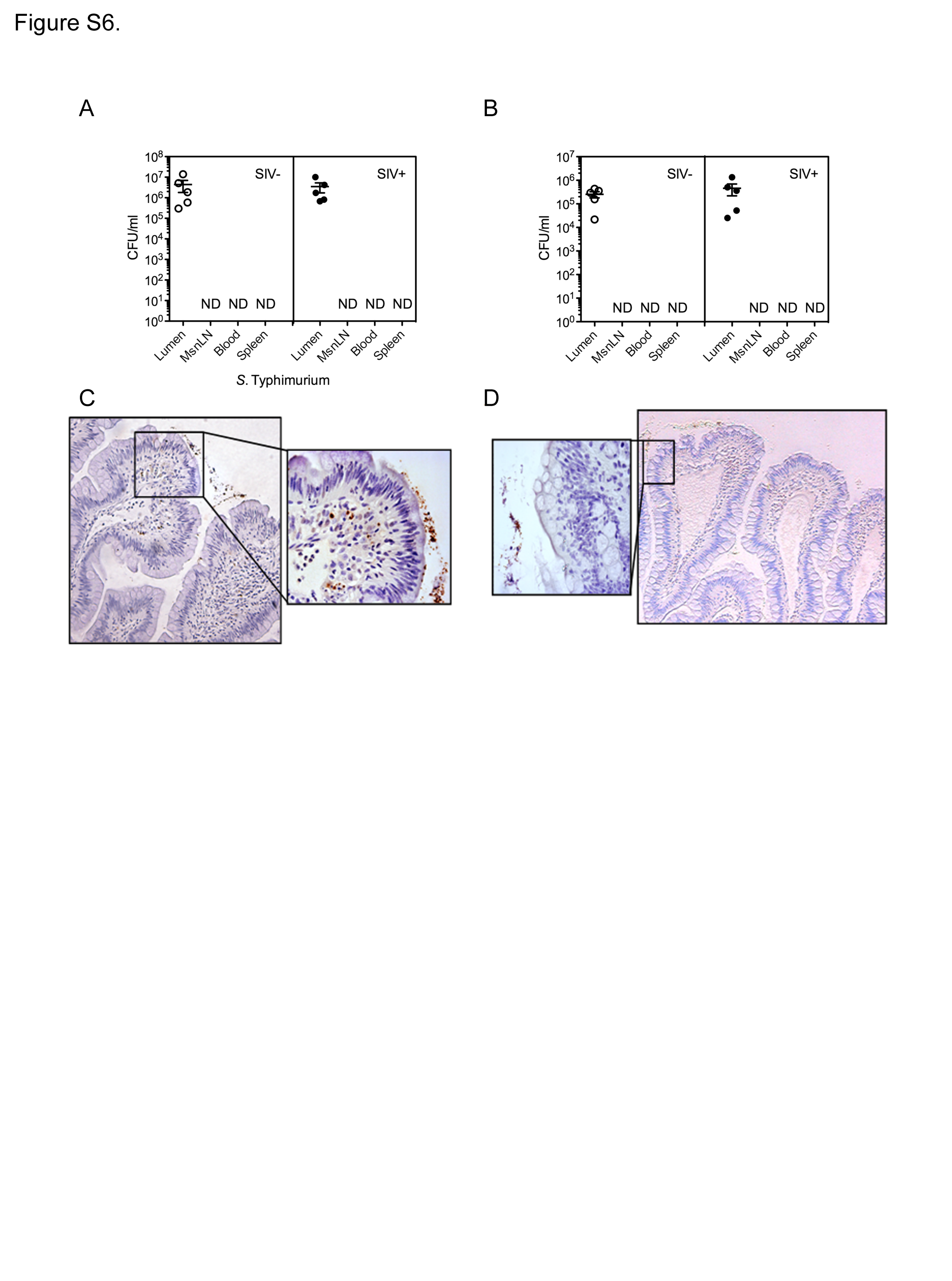

Supplement: Figure S6 — Absence of systemic translocation of S . Typhimurium or L. plantarum . Bacteriological assessments of live (A) S. Typhimurium and (B) L. plantarum were performed on the intestinal luminal content and sites peripheral to the intestinal mucosa: mesenteric lymph node (MsnLN), blood, and spleen. Immunohistochemistry was used to visualize the localization of (C) S. Typhimurium and (D) L. plantarum in the intestinal lumen and in the lamina propria of ileal loops. Representative images are shown at 20× magnification with insets shown at 60× magnification. (TIF) [file ppat.1004311.s006.tif]

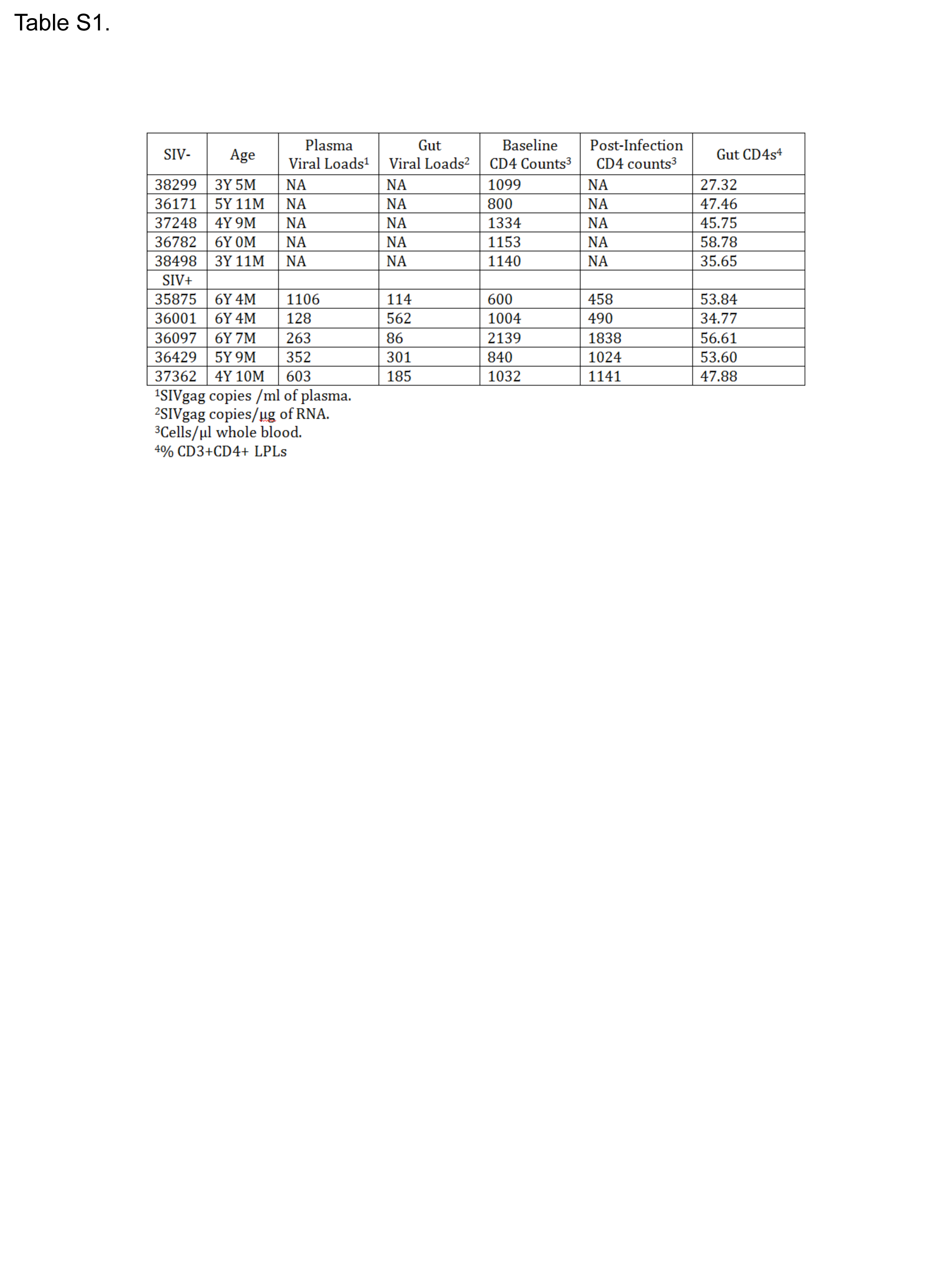

Supplement: Table S1 — Animal data and clinical parameters. (TIF) [file ppat.1004311.s007.tif]
